# Focal ischemic myocardial fibrosis assessed by late gadolinium enhancement cardiovascular magnetic resonance in patients with hypertrophic cardiomyopathy

**DOI:** 10.1186/s12872-024-03859-2

**Published:** 2024-04-09

**Authors:** Yang Zhi, Fu-dan Gui, Meng Xue, Yi-tian Long, Wen Miao, You Yi, Liang-chao Gao, Fu Bing, Shu-yue Pan

**Affiliations:** 1 Department of Radiology, Chengdu Fifth People’s Hospital, 33# Ma Shi Street, Chengdu, 611130 China; 2 Department of Cardiology, Chengdu Fifth People’s Hospital, 33# Ma Shi Street, Chengdu, 611130 China; 3 Department of Rheumatology and Immunology, Chengdu Fifth People’s Hospital, Chengdu, China

**Keywords:** Hypertrophic cardiomyopathy, Fibrosis, Late gadolinium enhancement, Cardiac magnetic resonance imaging

## Abstract

**Background:**

In patients with hypertrophic cardiomyopathy (HCM), ischemic myocardial fibrosis assessed by late gadolinium enhancement (I-LGE) using cardiovascular magnetic resonance (CMR) have been reported. However, the clinical significance of I-LGE has not been completely understood. We aim to evaluate the I-LGE differ phenotypically from HCM without LGE or nonischemic myocardial fibrosis assessed by late gadolinium enhancement (NI-LGE) in the left ventricle (LV).

**Methods:**

The patients with HCM whom was underwent CMR were enrolled, using cine cardiac magnetic resonance to evaluate LV function and LGE to detect the myocardial fibrosis. Three groups were assorted: 1) HCM without LGE; 2) HCM with LGE involved the subendocardial layer was defined as I-LGE; 3) HCM with LGE not involved the subendocardial layer was defined as NI-LGE.

**Results:**

We enrolled 122 patients with HCM in the present study. LGE was detected in 58 of 122 (48%) patients with HCM, and 22 (18%) of patients reported I-LGE. HCM with I-LGE had increased higher left ventricular mass index (LVMI) (*P* < 0.0001) than HCM with NI-LGE or without LGE. In addition, HCM with I-LGE had a larger LV end- systolic volume (*P* = 0.045), lower LV ejection fraction (LVEF) (*P* = 0.026), higher LV myocardial mass (*P* < 0.001) and thicker LV wall (*P* < 0.001) more than HCM without LGE alone. The I-LGE were significantly associated with LVEF (OR: 0.961; *P* = 0.016), LV mass (OR: 1.028; *P* < 0.001), and maximal end-diastolic LVWT (OR: 1.567; *P* < 0.001). On multivariate analysis, LVEF (OR: 0.948; *P* = 0.013) and maximal end-diastolic LVWT (OR: 1.548; *P* = 0.001) were associated with higher risk for I-LGE compared to HCM without LGE. Noticeably, the maximal end-diastolic LVWT (OR: 1.316; *P* = 0.011) was the only associated with NI-LGE compared to HCM without LGE.

**Conclusions:**

I-LGE is not uncommon in patients with HCM. HCM with I-LGE was associated with significant LV hypertrophy, extensive LGE and poor LV ejection fraction. We should consider focal ischemic myocardial fibrosis when applying LGE to risk stratification for HCM.

## Introduction

Hypertrophy cardiomyopathy (HCM) is a common hereditary cardiac disease in adults characterized by myocardial hypertrophy in the absence of abnormal loading conditions or systemic disease [1]. Most patients with HCM have a variable degree of myocardial fibrosis, and myocardial fibrosis is associated with worse outcomes [2–4]. In addition, previous studies have adequately described focal replacement myocardial fibrosis as a result of myocardial ischemia and microvascular dysfunction in patients with HCM [5–7]. Furthermore, myocardial fibrosis can be divided into several subgroups according to whether fibrosis involves the subendocardial layer, and these subgroups include nonischemic myocardial fibrosis and ischemic myocardial fibrosis [8, 9]. At present, the characteristics of focal myocardial fibrosis in patients with HCM remain unclear.

Late gadolinium enhancement (LGE) on cardiovascular magnetic resonance (CMR) is considered to be a promising modality to assess the location and quantization of myocardial fibrosis, which allows noninvasive detection technology in vivo [10, 11]. In addition, LGE-CMR has been routinely used to identify myocardial fibrosis in patients with HCM, as manifested by two major distribution patterns of LGE: intramural LGE or right ventricular insertion point LGE [8]. However, studies that have described the clinical significance of ischemic myocardial fibrosis in patients with HCM are scarce thus far [12, 13]. Moreover, there are no available data to demonstrate the effects of different distribution patterns of myocardial fibrosis on left ventricular function in HCM patients.

Here, the purpose of this study is to characterize ischemic myocardial fibrosis using LGE-CMR and to compare these patterns in patients with HCM. Furthermore, we sought to investigate the impact of ischemic myocardial fibrosis on LV function in patients with HCM.

## Materials and methods

### Study patients

We included patients with HCM (*n* = 122) who underwent LGE-CMR at Chengdu Fifth People’s Hospital (Chengdu, China) between 2015 and 2022. Patients who were diagnosed with HCM and who met the following inclusion criteria were included: (1) CMR demonstration of LV hypertrophy (maximal end-diastolic left ventricular wall thickness ≥ 15 mm or ≥ 13 mm in patients with a family history of HCM); (2) HCM patients aged ≥ 18 years; and (3) absence of other diseases that could result in LV hypertrophy. The major exclusion criteria were as follows: (1) patients with significant coronary disease and coronary artery stenosis ≥ 50%; (2) LV septal resection or ablation therapy; (3) patients with severe valvular disease, cardiac amyloidosis, congenital heart disease, or CMR images that were poor and not analyzed; and (4) patients with a history or a medical record of myocardial infarction (MI). Furthermore, clinical data, including age, height, weight, laboratory data, and other baseline characteristics, were obtained from the electronic medical records. This study was approved by the Institutional Review Board of the Chengdu Fifth People’s Hospital (2020–036-01).

### CMR imaging

CMR imaging was performed with a MAGNETOM Vida 3 T scanner (Siemens, Erlangen, Germany) or an Achieva 1.5 T scanner (Philips, Best, the Netherlands) using a body coil. The CMR protocol included cine and LGE from the base of the heart to the apex, using retrospective ECG-gate and breath-hold. For short-axis 4-chamber, and 3-chamber cine images, the sequence parameters by the 1.5-T scanner were: field-of-view: 320 mm, repetition/echo time: 3.4/1.69 ms, flip angle: 60°, matrix: 192 × 192, slice thickness: 8 mm with no gap, and phases: 30. The sequence parameters by the 3.0-T scanner were: field-of-view: 420 mm, repetition/echo time: 39.12/1.43 ms, flip angle: 80°, matrix: 256 × 199, slice thickness: 8 mm with no gap, and phases: 25. LGE images were obtained 10–15 min after intravenous administration of a 0.1 mmol/kg gadobenate dimeglumine injection (MultiHance; Bracco) using a phase-sensitive inversion recovery (PSIR) sequence in the same position as the cine images. The parameters for LGE images were as follows: for a 1.5 T scanner, field-of-view: 320 mm, repetition/echo time: 6.1/3.0 ms, flip angle: 25°, matrix: 200 × 152, and slice thickness: 8 mm with no gap; for a 3.0 T scanner, field-of-view: 420 mm, repetition/echo time: 740/1.06 ms, flip angle: 40°, matrix: 256 × 144, and slice thickness: 8 mm with no gap. The inversion time was adjusted by TI scout for contrast optimization.

### CMR analysis

LV and RV function, including left ventricular ejection fraction (LVEF), left ventricular end-diastolic volume (LVEDV), left ventricular end-systolic volume (LVESV), left ventricular stroke volume (LVSV), LV mass, right ventricular ejection fraction (RVEF), right ventricular end-diastolic volume (RVEDV), right ventricular end-systolic volume (RVESV), and right ventricular stroke volume (RVSV) analysis were performed using cvi42 software (v. 5.15.4, Circle Cardiovascular Imaging, Calgary, Canada) by a radiologist with 4 years of experience in CMR who was blinded to the clinical data of each patient. The maximal end-diastolic left ventricular wall thickness (LVWT) was assessed at end-diastole in the short axis cine view. Furthermore, for LV and RV functional analysis, the epicardium and endocardium were manually traced on short-axis cine images, including the papillary muscles. The visual assessment of LGE was undertaken by two researchers blinded to the clinical information, followed by classification when LGE was present, and the LGE was classified as either I-LGE (LGE involved the subendocardial layer) or NI-LGE (LGE not involved the subendocardial layer). If there was discordance between the diagnosis of the LGE classification between the two researchers, they discussed the findings with each other and reached a consensus. If the HCM patients with visible LGE, LGE was calculated as a percentage of total LV mass and defined as myocardium 5 standard deviations (SD) above the mean of remote area of myocardium without visual LGE. We also provided a score of zero to assess the extent of LGE of patients with no visible LGE.

### Statistical analysis

Statistical analyses were performed using GraphPad Prism 7 (GraphPad Software, Inc). The normality of the data was assessed by the D’Agostino & Pearson normality test. Continuous data were represented by means ± SD, and nonnormally distributed data were represented as medians (with interquartile range). Comparison of the CMR parameters between the subgroups of HCM was evaluated using one-way analysis of variance (ANOVA) with LSD tests for continuous variables or the Kruskal‒Wallis test, as appropriate. Univariate logistic regression analyses were used to explored the associations between the patterns of the LV LGE and LV structure and function parameters, and each parameter with a *P* < 0.05 was entered into the multivariable logistic regression analysis to calculate the LV function parameters associated with I-LGE or NI-LGE independently. In addition, correlations between LGE extent and LV function parameters were tested using Spearman correlation analysis. A two-tailed *P* value < 0.05 was considered statistically significant.

## Results

### Study population

**Fig. 1:**
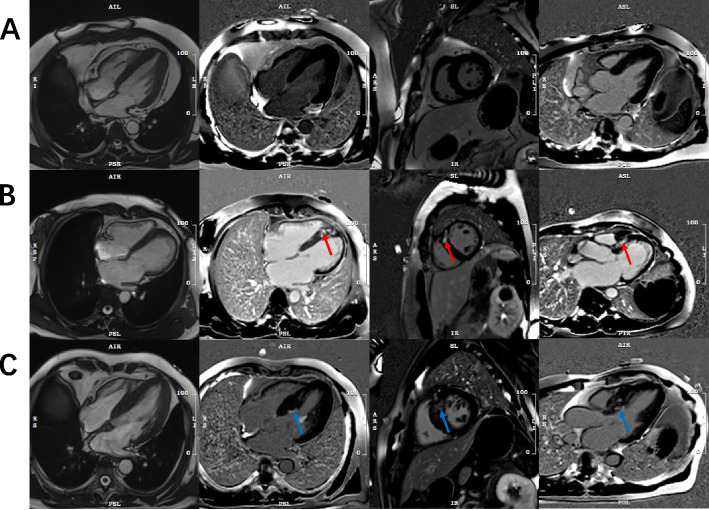
Examples of focal myocardial fibrosis in patients with HCM. The case shows an HCM patient without LGE (**A**). CMR images of patients with nonischemic myocardial fibrosis (red arrow) detected by LGE (**B**). The case showed the presence of LGE and involved the subendocardial layer (blue arrow), which was defined as ischemic myocardial fibrosis (**C**). LGE, late gadolinium enhancement

Among the patients with HCM, 22 patients (18%) had I-LGE, 36 patients (30%) had LGE confined to NI-LGE, and 64 patients (52%) did not have LGE (Fig. 1). The mean age of HCM patients with I-LGE was 57.00 ± 12.90 years old, and 68% were male. Furthermore, the mean age and sex were similar among the three groups (all *P* > 0.05). In the HCM with I-LGE group, 2 patients (10%) had diabetes, and 10 patients (48%) had hypertension. HCM comorbidities, such as diabetes and hypertension, were similar among the three groups (all *P* > 0.05). Table 1 depicts the clinical characteristics stratified by the presence or absence of I-LGE. The HCM patients with I-LGE had more N-terminal prohormone of brain natriuretic peptide (NT-proBNP) than the patients without LGE (4857 pg/ml vs. 1195 pg/ml; *P* = 0.005). In addition, no significant differences were found among the three groups with respect to the other baseline characteristics.

**Table 1:** Baseline characteristic of HCM patients based on the presence of I-LGE on CMR

	HCM with I-LGE (Group 1, *n* = 22)	HCM with NI-LGE (Group 2, *n* = 36)	HCM without LGE (Group 3, *n* = 64)	ANOVA (*P*-value)
**Baseline characteristics**				
Age, (years ± SD)	57.00 ± 12.90	58.47 ± 15.20	60.11 ± 15.32	0.320
Males, *n* (%)	15 (68.18%)	20 (55.55%)	33 (51.56%)	0.399
Height, cm	161.80 ± 7.21	159.9 ± 7.75	160.4 ± 8.33	0.807
Weight, Kg	65.7 ± 9.92	62.61 ± 11.33	65.80 ± 13.33	0.470
Hypertension, *n* (%)	10 (47.61%)	16 (50.00%)	36 (62.06%)	0.380
Diabetes, *n* (%)	2 (10.00%)	5 (25.62%)	14 (24.13%)	0.320
Alcohol, *n* (%)	7 (35.00%)	7 (21.87%)	15 (25.86%)	0.574
Smoker, *n* (%)	8 (42.10%)	10 (31.25%)	17 (29.31%)	0.579
Systolic blood pressure (mmHg)	124.40 ± 19.11	122.10 ± 15.27	123.70 ± 14.80	0.839
Diastolic blood pressure (mmHg)	75,43 (67.00, 78.00)	67.88 ± 10.75	71.17 ± 9.52	0.181
Heart rate (bpm)	74.91 ± 17.66	74.08 ± 13.86	73.79 ± 14.51	0.974
**Laboratory data**				
Hematocrit (%)	41.56 ± 5.35	40.51 ± 6.88	39.56 ± 7.19	0.804
Blood glucose (mg/dL)	7.63 (2.28, 8.19)	6.43 (4.79, 6.39)	7.18 (5.17, 7.81)	0.337
Hemoglobin A1c (mg/dL)	6.42 (5.50, 6.50)	5.81 (5.10, 6.15)	6.37 (5.67, 7.02)	0.086
C-reactive protein (mg/dL)	13.08 (1.17, 4.00)	16.46 (1.00, 6.35)	9.98 (1.47, 7.5)	0.347
NT-proBNP (pg/mL)	4857 (1101, 6596)	3078 (289.3, 3078)	1195 (155.9, 1093)	**0.003**
BUN (umol/L)	7.00 (5.31, 7.04)	7.24 (4.93, 8.03)	7.06 (4.85, 9.07)	0.920
Serum creatinine (umol/L)	79.36 (62.00, 87.35)	123.20 (63.95, 103.90)	72.71 (60.38, 78.25)	0.117
Total cholesterol (mmol/L)	4.80 ± 0.83	4.79 ± 1.36	4.52 ± 1.21	0.262
Triglyceride (mmol/L)	3.44 (0.98, 4.73)	2.66 (1.18, 2.78)	2.19 (1.09, 2.63)	0.747
HDL-cholesterol (mmol/L)	1.20 (0.94, 1.32)	1.18 (1.00, 1.41)	1.38 (0.90, 1.36)	0.977
Troponin I (ng/mL)	0.23 (0.001, 0.52)	0.15 (0.001, 0.01)	0.04 (0.001, 1.06)	0.395
Myoglobin (ng/mL)	73.34 (37.47, 62.00)	65.81 (35.31, 65.40)	50.47 (33.89, 57.49)	0.803
CK-MB (U/L)	14.63 (10.25, 16.50)	12.50 (9.00, 14.50)	12.86 (9.00, 15.25)	0.341

*HCM* hypertrophic cardiomyopathy, *I-LGE* ischemic myocardial fibrosis assessed by late gadolinium enhancement, *NI-LGE* nonischemic myocardial fibrosis assessed by late gadolinium enhancement, *CMR* cardiovascular magnetic resonance, *NT-proBNP* N-terminal prohormone of the brain natriuretic peptide, *CK-MB* creatine kinase-MB

### CMR findings

Table 2 shows the distribution among the three groups. The patients with HCM with I-LGE had significantly lower LVEF (*P* = 0.026) and higher LVESV (*P* = 0.045) than the patients HCM without LGE; however, there was no significant difference in LVEF and LVESV between the patients with I-LGE versus those with NI-LGE. In addition, LV mass was significantly higher in the HCM patients with I-LGE (*P* < 0.001) or NI-LGE (*P* = 0.024) than in the HCM patients without LGE; however, there was no significant difference between the patients with I-LGE and NI-LGE (*P* = 0.122). Compared with the patients without LGE, LV mass index progressively increased in the I-LGE and NI-LGE patients: 1.41 ± 0.47, 18.67 ± 0.27, and 1.01 ± 0.18, respectively (overall *P* < 0.001; I-LGE patients *P* < 0.05 vs. each of the other groups). Importantly, maximal end-diastolic LVWT was greater in the patients with HCM with LGE confined to I-LGE than in the patients without LGE (21.00 ± 4.66 mm vs. 16.77 mm (15.00–18.00); *P* < 0.001); however, there was no significant difference between the patients with I-LGE versus those with NI-LGE (*P* = 0.415). Conversely, right ventricular (RV) function, including RVEF, RVEDV, RVESV and RVSV, was virtually identical in the I-LGE patients, NI-LGE patients, and patients without LGE (all *P* > 0.05). In addition, there was no significant difference in the number of LVOT obstruction among the three groups (all *P* > 0.05).

**Table 2:** CMR features of the HCM patients depending on the presence and location of LGE on CMR

	HCM with I-LGE (Group 1, *n* = 22)	HCM with NI-LGE (Group 2, *n* = 36)	HCM without LGE (Group 3, *n* = 64)	ANOVA (*P*-value)	*P* 1 vs. 2	*P* 1 vs. 3	*P* 2 vs. 3
**CMR findings**							
LVEF (%)	69.14 (47.57, 73.58)	69.45 (63.30, 78.93)	70.92 (67.20, 79.04)	**0.032**	0.304	**0.026**	0.987
LVEDV (mL)	130.60 (90.89, 151.90)	117.10 (96.56, 129.50)	112.50 (83.57, 131.70)	0.167	0.991	0.193	> 0.999
LVESV (mL)	49.79 (28.90, 62.61)	38.52 (19.20, 45.19)	35.23 (19.68, 38.15)	0.052	0.313	**0.045**	> 0.999
LVSV (mL)	75.33 ± 27.16	78.60 ± 19.36	77.24 ± 24.37	0.876	0.865	0.9424	0.958
LV mass (g)	170.8 (136.1, 202.6)	128.50 (99.30, 164.3)	111.6 (84.74, 129.5)	** < 0.001**	0.122	** < 0.001**	**0.024**
LV mass index (g/m2)	1.41 ± 0.47	1.16 ± 0.27	1.01 ± 0.18	** < 0.001**	**0.006**	** < 0.001**	**0.023**
Max LVWT (mm)	19.50 (17.75, 25.00)	18.67 (17.00, 20.00)	16.77 (15.00, 18.00)	** < 0.001**	0.415	** < 0.001**	**0.003**
RVEF (%)	64.30 (41.32, 69.33)	60.39 (55.57, 68.29)	58.32 (50.42, 68.05)	0.608	> 0.999	> 0.999	0.966
RVEDV (mL)	99.07 (59.71, 131.7)	95.29 (69.12, 115.2)	97.33 (72.41, 114.8)	0.712	> 0.999	> 0.999	> 0.999
RVESV (mL)	41.48 (28.53, 49.79)	39.09 (26.05, 46.78)	40.09 (27.26, 151.4)	0.390	0.561	> 0.999	0.980
RVSV (mL)	25.09 ± 57.25	56.20 ± 21.48	57.24 ± 17.92	0.9678	0.980	> 0.999	0.967
LVOT obstruction (n %)	10 (45.45%)	16 (19.88%)	24 (54.54%)	0.711	-	-	-
LGE extent (%)	13.77 ± 12.22	6.63 ± 3.60	0	** < 0.001**	**0.267**	** < 0.001**	** < 0.001**

*HCM* hypertrophic cardiomyopathy, *LGE* late gadolinium enhancement, *CMR* cardiovascular magnetic resonance, *LVEF LV* left ventricular, *LVEF* left ventricular ejection fraction, *LVEDV* left ventricular end-diastolic volume, *LVESV* left ventricular end-systolic volume, *LVSV* left ventricular stroke volume, *RVEF* right ventricular ejection fraction, *RVEDV* right ventricular end-diastolic volume, *RVESV* right ventricular end-systolic volume, *RVSV* right ventricular stroke volume, *LVWT* left ventricular wall thickness, *LVOT* left ventricular outflow tract

### Correlations

As presented in Table 3, the patterns of I-LGE were association with LVEF, LV mass, and maximal end-diastolic LVWT (all *P* < 0.05), but not with LVEDV, LVESV, LVSV, and LVCO (all *P* > 0.05). Multivariate logistic regression analysis showed that LVEF and maximal end-diastolic LVWT were associated with a higher risk for I-LGE than HCM without LGE (all *P* < 0.05), whereas LV mass (*P* = 0.254) was not associated with NI-LGE. Noticeably, the maximal end-diastolic LVWT (OR: 1.316; 95% CI: 1.064–1.627; *P* = 0.011) was the only associated with NI-LGE compared to HCM without LGE.

**Table 3:** Univariate and multivariate logistic regression analysis for I-LGE in HCM patients

	Univariate analysis	Multivariate analysis
Variable	OR (95% CI); *P*-value	OR (95% CI); *P*-value
LVEDV	1.007 (0.996–1.019); *P* = 0.208	-
LVESV	1.012 (0.998–1.027); *P* = 0.083	-
LVSV	0.996 (0.976–1.018); *P* = 0.739	-
LVEF	0.961 (0.930–0.993); *P* = 0.016	0.948 (0.910–0.989); *P* = 0.013
LVCO	0.980 (0.720–1.334); *P* = 0.896	-
LV mass	1.028 (1.015–1.042); *P* < 0.001	1.010 (0.993–1.027); *P* = 0.254
The maximal end-diastolic LVWT	1.567 (1.288–1.908); *P* < 0.001	1.548 (1.204–1.990); *P* = 0.001

*HCM* hypertrophic cardiomyopathy, *I-LGE* ischemic myocardial fibrosis assessed by late gadolinium enhancement, *LV* left ventricular, *LVEF* left ventricular ejection fraction, *LVEDV* left ventricular end-diastolic volume, *LVESV* left ventricular end-systolic volume, *LVSV* left ventricular stroke volume, *LVWT* left ventricular wall thickness

In addition, HCM patients with extensive LGE had significantly higher LVESV (*r* = 0.212, *P* = 0.019), LV mass (*r* = 0.406, *P* < 0.001), LV mass index (*r* = 0.399, *P* < 0.001), and the maximal end-diastolic LVWT (*r* = 0.441, *P* < 0.001). Besides, the LGE extent was negatively associated with LVEF (*r* = -0.250, *P* = 0.006) in patients with HCM. Moreover, the LGE extent were related to the levels of NT-proBNP and showed a trend with increasing NT-proBNP (*r* = 0.399, *P* = 0.001).

## Discussion

The main findings of this study demonstrate that HCM patients with ischemic-like LGE confined to I-LGE were present on CMR in 18% of cases. HCM with I-LGE was associated with larger LVESV, increased LV mass and LV mass index, decreased LVEF and thicker maximal end-diastolic LVWT. Importantly, compared to HCM patients without LGE, HCM patients with thickening of maximal end-diastolic LVWT and larger LVESV were associated with a higher risk of I-LGE.

LGE-CMR is considered a noninvasive tool for the assessment of the presence and location of focal myocardial fibrosis in patients with HCM [14]. In our study, LGE was present in 48% of patients, which is in line with previous studies in patients with HCM [15]. Although previous studies have mainly demonstrated that LGE-CMR is associated with adverse ventricular remodeling and is a marker of serious cardiac complications in HCM, there is a lack of studies classifying LGE [16]. In this study, two patterns of LGE were found in HCM: involvement of the subendocardial layer and LGE that is focal patchy in the mid-wall. The pattern of nonischemic LGE of the mid-wall is known to be typical LGE, which is a characteristic feature of HCM [17]. However, we defined the LGE pattern involvement of the subendocardial layer as I-LGE, which was detected in 18% of patients with HCM. Importantly, few studies have noted the distribution of I-LGE in HCM. Furthermore, the clinical significance of I-LGE in the context of HCM remains uncertain.

In the current study, we observed that LGE preferentially involved the basal and midventricular septum and anterior free wall in HCM [18]. We further evaluated the LGE patterns of different types of patients with HCM. Patients with HCM and LGE were roughly divided into I-LGE and NI-LGE groups. Our results confirmed that I-LGE is related to impaired LV mechanics with decreased LVEF. Furthermore, HCM with I-LGE showed higher LV mass, maximal end-diastolic LVWT, and LV mass index than HCM patients without LGE, whereas the LV mass index was higher in patients with I-LGE than in those with LGE confined to the NI-LGE. HCM with a higher LV mass index is associated with more advanced ventricular remodeling and influences LV systolic mechanics [19]. In addition, HCM with a high LV mass was associated with LGE progression [20]. Therefore, it is necessary to screen and classify LGE in patients with severe myocardial hypertrophy.

CMR measurements of LVESV, LVEDV, LV mass, end-diastolic LVWT, and LGE have proven to be valuable imaging tools for risk stratification of HCM [16, 21]. The results suggest that the participants with maximal LV mass index and end-diastolic LVWT value correlated with I-LGE. Meanwhile, as a result of LV wall thickening and myocardial remodeling, LV mass increases [22, 23]. In addition, a large LVESV, low LVEF and high LV mass were associated with I-LGE. Of note, maximal end-diastolic LVWT was powerfully associated with I-LGE and NI-LGE in patients with HCM, independent of LVESV, LVEDV, LV mass, and LV mass index, suggesting that severe myocardial hypertrophy can lead to I-LGE. This suggestion shows that the differences in LGE patterns between HCM appeared to be driven primarily by the degree of LVMT thickness. In line with our study, previous studies have demonstrated that LV hypertrophy and disarray of the myocardium in patients with HCM may lead to increased LVWT thickness, and LVWT is significantly associated with LGE [24–26]. Furthermore, our data demonstrate that LVESV is also linked to I-LGE. This may be due to damage to the longitudinal myocardium under the endocardium in HCM patients with I-LGE, which leads to a decrease in cardiac systolic function and an increase in LVESV.

Moreover, we showed higher NT-proBNP levels in HCM patients with I-LGE. Consistent with our findings, previous studies have shown a relationship between NT-proBNP and LGE progression [27, 28]. In addition, NT-proBNP levels have been found to be associated with the LGE extent. NT-proBNP may be a potential marker for extensive LGE extent in HCM. Moreover, patients with increased NT-proBNP levels were associated with an increased risk of cardiac death [29, 30]. This suggests that I-LGE is a sign of advanced cardiomyopathy, which could explain the higher level of NT-proBNP than NI-LGE in HCM [31].

Notably, a recent study demonstrated that HCM patients with I-LGE had higher LGE extent than those with NI-LGE, and this was in line with our study [32]. Moreover, previous studies showed that, the type of I-LGE, rather than LGE extent, was association with adverse outcomes among the HCM patients with LGE < 15% LV mass [32, 33]. It was noted that HCM patients who with the type of I-LGE, the LV myocardial was significantly remodeled, and the degree of LV remodeling is severe, with the extensive LGE. Given the I-LGE was showed in 18% of HCM patients in present study, identification of the type of I-LGE using LGE-CMR may provide risk stratification.

Currently, the mechanism of I-LGE in patients with HCM remains unclear. On the one hand, it was mainly explained by the fact that hypertrophic myocardium and the disordered arrangement of fibers led to subendocardial myocardial ischemia. Previous studies have suggested that LGE is driven by myocardial ischemia in HCM, and impaired myocardial energetics caused by ischemia may contribute to I-LGE progression [20, 34]. On the other hand, it has been reported that LV wall thickness is significantly related to small vessel disease in patients with HCM [35]. Consequently, we speculate that injury to these small vessels may lead to I-LGE in HCM, but further studies are needed. Moreover, patients with HCM have increased levels of coronary microvascular dysfunction and reduced subendocardial flow, eventually leading to myocardial ischemia [36, 37]. Chronic and recurrent myocardial ischemia can contribute to I-LGE in patients with HCM [38]. This could explain why HCM with I-LGE had a higher LV mass and greater LV wall thickness in the present study. However, no studies have systematically assessed the contribution of myocardial ischemia to I-LGE thus far.

## Limitations

First, our study was a retrospective, observational study that possibly includes selection bias. In addition, myocardium with a lower degree of focal fibrosis may not be detected by the visual assessment of LGE-CMR, and imaging with T1 mapping was not used to evaluate diffuse fibrosis in this article. Furthermore, although genes play an important role in myocardial fibrosis and the clinical prognosis in HCM, genetic testing was not routinely used in our study. Genetic testing is expensive. In addition, the use of scanners with different field strengths to obtain CMR images is a potential limitation. Another limitation of this study was that it was a cross-sectional study, which requires continuous follow-up of this cohort to further improve our understanding of the prognostic value of I-LGE in HCM.

## Conclusion

In patients with HCM, I-LGE is not uncommonly detected by LGE-CMR despite a preserved left ventricular function. In addition, I-LGE was associated with adverse cardiac remodeling, increased LV mass index, and thickening maximal end-diastolic LVWT in patients with HCM. Furthermore, LVWT appears to play a role in the development of I-LG in HCM. Future studies will be required to assess the potential prognostic value of I-LGE and may help with risk stratification in patients with HCM.

## Data Availability

The datasets used and/or analysed during the current study available from the corresponding author on reasonable request.

## Abbreviations

HCM: Hypertrophic cardiomyopathy
I-LGE: Ischemic myocardial fibrosis assessed by late gadolinium enhancement
CMR: Cardiovascular magnetic resonance
NI-LGE: Nonischemic myocardial fibrosis assessed by late gadolinium enhancement
LV: Left ventricle
LVMI: Left ventricular mass index
LVEF: Left ventricular ejection fraction
LVEDV: Left ventricular end-diastolic volume
LVESV: Left ventricular end-systolic volume
LVSV: Left ventricular stroke volume
RVEF: Right ventricular ejection fraction
RVEDV: Right ventricular end-diastolic volume
RVESV: Right ventricular end-systolic volume
RVSV: Right ventricular stroke volume
LVWT: Left ventricular wall thickness
SD: Standard deviation
